# Fermented dairy intake and markers of musculoskeletal ageing on the island of Ireland: insights from the Northern Ireland Cohort for the Longitudinal Study of Ageing (NICOLA) and The Irish Longitudinal Study on Ageing (TILDA)

**DOI:** 10.1007/s00394-026-04150-0

**Published:** 2026-11-04

**Authors:** Dominic N. Farsi, Nicola A. Ward, Charlotte E. Sterling, Angela Scott, Gareth J. McKay, Claire T. McEvoy, Bernadette McGuinness, Paul D. Cotter, Orla O’Sullivan

**Affiliations:** 1 Food Biosciences, Teagasc Food Research Centre, Moorepark, Co. Cork, Ireland; 2 VistaMilk, Co. Cork, Ireland; 3 Centre for Public Health, School of Medicine, Dentistry and Biomedical Sciences, Queen’s University Belfast, Belfast, UK; 4 Department of Nutritional Sciences, King’s College London, London, UK; 5 APC Microbiome Ireland, University College Cork, Co. Cork, Ireland

**Keywords:** Fermented dairy, Ageing, Physical function, Musculoskeletal health, Handgrip strength, Gut–muscle axis

## Abstract

**Purpose:**

Fermented dairy foods may support healthy musculoskeletal ageing via high-quality protein and gut-muscle axis modulation. Epidemiological evidence linking fermented dairy foods with physical function in older adults is limited. We examined associations between fermented dairy intake and physical function in older adults in Ireland using two independent population-based cohorts, to assess the consistency of associations across differing dietary assessment approaches.

**Methods:**

Cross-sectional analyses of data from the Northern Ireland Cohort for the Longitudinal Study of Ageing (NICOLA; n = 2791; mean age 64 ± 9 years) and The Irish Longitudinal Study on Ageing (TILDA; n = 3999; mean age 66 ± 9 years) was performed. Fermented dairy intake was calculated from cohort-specific food frequency questionnaires (FFQs) with outcomes including muscle strength, frailty and functional limitations. Multivariable linear and logistic regression models were applied, adjusting for demographic and health-related covariates.

**Results:**

In NICOLA, fermented dairy intake was not associated with physical function outcomes after confounder adjustment. Associations observed in crude models were attenuated after adjustment, with no dose–response relationships. In TILDA, participants in the highest tertile of fermented dairy intake had higher odds of low grip strength compared with those in the lowest tertile (OR = 2.71; 95% CI: 1.12, 6.88; *p* = 0.030). However, analysis of individual fermented dairy foods indicated that this association was driven by yoghurt intake and was attenuated after covariate adjustment.

**Conclusions:**

Across two independently analysed cohorts, fermented dairy intake was not consistently associated with physical function after confounder adjustment. There is a need for longitudinal and interventional studies to better determine the role of fermented dairy foods in musculoskeletal ageing.

**Supplementary Information:**

The online version contains supplementary material available at https://doi.org/10.1007/s00394-026-04150-0.

## Introduction

Ageing can be accompanied by sarcopenia, characterised by the loss of muscle mass, strength, and physical performance, which is increasingly recognised as a major contributor to frailty, loss of independence, and adverse health outcomes in older adults [1, 2]. Beyond physical consequences, declining skeletal muscle health has also been associated with increased risk of cognitive impairment [3, 4]. Accordingly, strategies to preserve skeletal muscle health and mitigate age-related functional decline represent an important public health priority.

Among modifiable factors, diet, particularly protein intake, plays a central role in the regulation of skeletal muscle mass and function [5]. Adequate dietary protein is required to stimulate muscle protein synthesis and maintain skeletal muscle tissue, yet ageing is associated with anabolic resistance, defined as a reduced sensitivity of skeletal muscle to anabolic stimuli such as dietary protein intake [6]. This blunted anabolic response necessitates not only sufficient total protein intake but also consideration of protein quality, amino acid composition, and distribution across meals [6–8]. Accordingly, optimisation of dietary protein intake is considered a cornerstone of strategies aimed at preserving skeletal muscle mass and function in older adults.

Within this context, protein-rich foods have received considerable attention, with dairy foods representing a key source of high-quality protein in many dietary patterns. Dairy foods provide a rich supply of essential amino acids alongside micronutrients relevant to musculoskeletal health [9]. However, dairy is a heterogeneous food group, and there is increasing interest in determining if specific subtypes may exert differential physiological effects. Fermented dairy foods, including yogurt and cheese, are of particular interest due to their complex food matrices, encompassing not only protein and micronutrients but also live microorganisms and fermentation-derived metabolites [10–12]. These characteristics suggest that fermented dairy products may confer additional physiological benefits beyond those attributable to protein content alone.

**Fig. 1:**
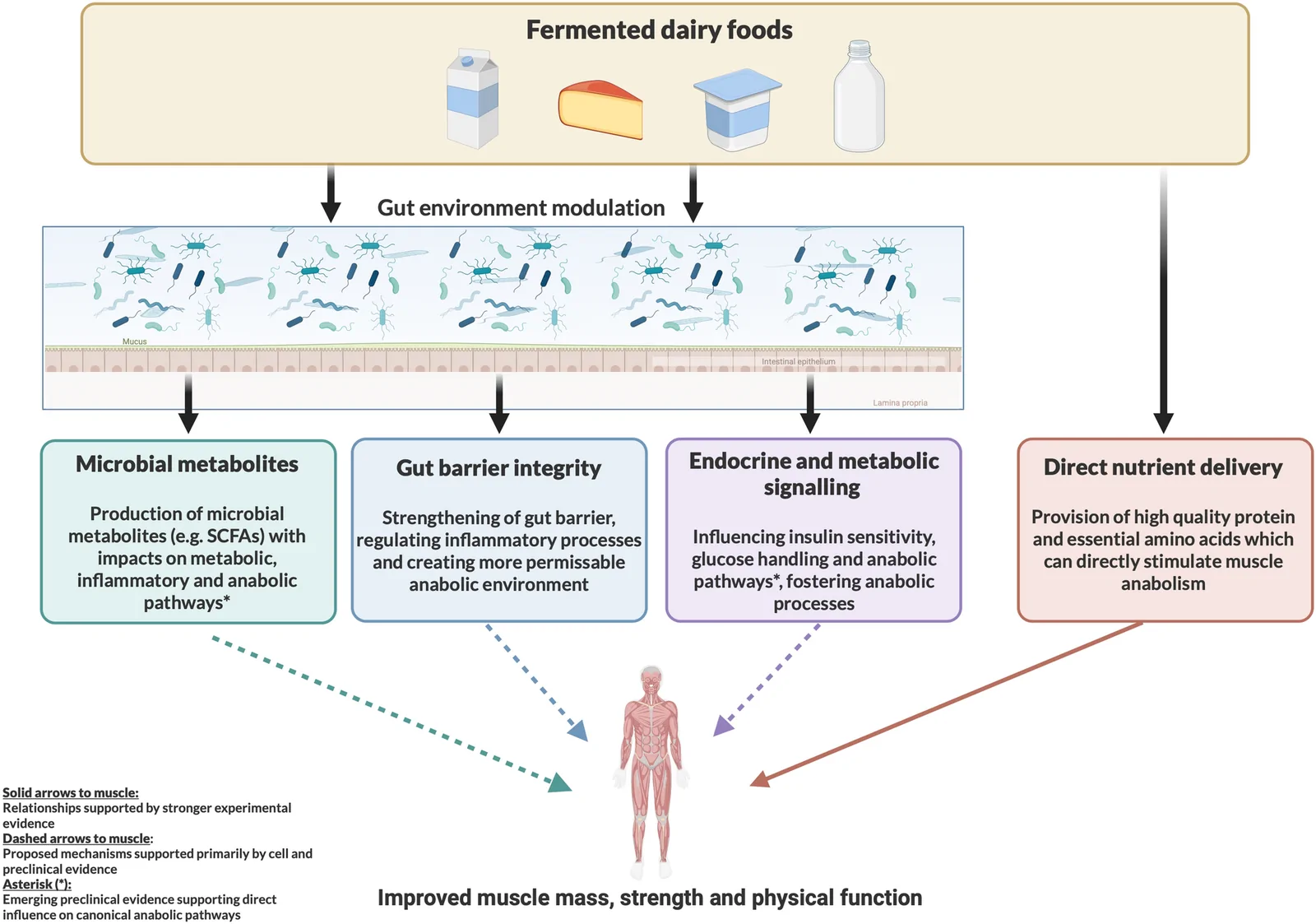
Conceptual framework illustrating potential pathways linking fermented dairy intake with musculoskeletal health

One potential pathway linking fermented dairy intake to skeletal muscle health is through interactions with the gut microbiome. Emerging evidence supports the concept of a gut-muscle axis, describing bidirectional interactions between the gut microbiome and skeletal muscle physiology. Through this axis, the gut microbiome may influence skeletal muscle physiology via the production of bioactive metabolites, modulation of gut barrier integrity and systemic inflammation, regulation of endocrine signalling pathways, and effects on nutrient bioavailability [13–15]. Conversely, host-related factors, including diet and physical activity, may shape gut microbial composition and function [16, 17]. Fermented dairy foods may modulate these pathways through both their intrinsic microbial content and bioactive compounds generated during fermentation [15], while also delivering high quality protein and a complete amino acid profile which may support muscle protein synthesis in the context of age-related anabolic resistance (Fig. 1).

Despite this strong biological plausibility, population-level evidence linking fermented dairy intake with functional outcomes remains limited. While certain fermented dairy foods, such as cottage cheese, are frequently promoted as beneficial for muscle health, these claims are often extrapolated from mechanistic insights rather than supported by robust epidemiological or interventional evidence [18]. To date, relatively few studies have examined associations between fermented dairy consumption and muscle strength, mobility, and/or frailty, and there is a lack of analyses across independent population-based cohorts to assess the consistency and generalisability of observed associations. Addressing this evidence gap is important given the growing interest in dietary strategies aimed at supporting healthy ageing, preserving physical function, and maintaining independence in older adults.

Therefore, the aim of the present study was to examine associations between fermented dairy intake and measures of physical function in older adults residing in Ireland. Data were drawn from two well-characterised population-based ageing cohorts, the Northern Ireland Cohort for the Longitudinal Study of Ageing (NICOLA) and The Irish Longitudinal Study on Ageing (TILDA), which include detailed information on dietary intake, physical function, and health-related factors [19–22]. Primary analyses were conducted in NICOLA and subsequently repeated in TILDA to examine whether similar patterns were observed in an independent cohort employing different dietary assessment methodologies. We hypothesised that higher fermented dairy intake would be associated with more favourable physical function and lower frailty in older adults.

## Methods

### Main analysis in NICOLA

#### Study population

Participants were drawn from Wave 1 of NICOLA (2013–2016), a longitudinal population study of community-dwelling adults ages ≥ 50 years living in Northern Ireland [20]. At Wave 1, 8,478 participants were recruited from a random, stratified sample of addresses derived from the General Practitioner Register to provide a nationally representative cohort. Data collection included a computer-assisted personal interview (CAPI), a self-completion questionnaire (SCQ), a food frequency questionnaire (FFQ) and a detailed health assessment providing objective measures of physical and cognitive function. Full details of the NICOLA study design and methodology have been described previously [20, 22], while the NICOLA study website provides details of accessible data through a searchable data dictionary (https://www.qub.ac.uk/sites/NICOLA/InformationforResearchers/#requesting-access-to-nicola-data-or-biological-samples-910951-1).

#### Dietary assessment and exposure definition

Of the 8,478 NICOLA Wave 1 participants, dietary intake was assessed and available for 2892 individuals at baseline using a semi-quantitative validated European Prospective Investigation into Cancer and Nutrition (EPIC)-Norfolk FFQ [23]. Participants reported habitual intake over the preceding year. Daily intakes (grams/day) for individual food items were derived using standard portion sizes and frequency responses. Total energy intake (kcal/day) was calculated using Nutritics software to determine energy and nutrient intakes [24]. Participants with implausible total energy intakes were excluded prior to analysis. Cut-offs were based on commonly used thresholds in nutritional epidemiology (females: < 500 or > 3500 kcal/day; males: < 800 or > 4200 kcal/day) [25]. This removed 101 participants, with 2791 remaining for the analysis.

Total fermented dairy intake (g/day) was estimated by summing the available FFQ items classified a priori as fermented dairy foods, including yoghurt, cheese, and other relevant fermented dairy items where available (e.g. cottage cheese). The resulting exposure represents an operational definition based on the fermented dairy food items captured by the NICOLA cohort FFQ. The exposure was analysed as a continuous variable (per 50 g/day increase in intake) and additionally categorised into tertiles (low, medium, high) based on the cohort distribution to allow for the assessment of potential non-linear associations.

#### Outcome measures

##### Muscle strength

Participants were invited to attend a health assessment at the Wellcome Trust-Wolfson Northern Ireland Clinical Research Facility (NICRF) located at the Belfast City Hospital, while a nurse-led home assessment was offered to respondents who were unable or unwilling to attend the NICRF. All assessments were undertaken by research nurses and research assistants who received comprehensive training in the methodologies [22]. Hand grip strength (HGS) was measured using a Baseline® Hydraulic Hand Dynamometer (Performance Health, UK). Participants held the dynamometer with the dial facing away, keeping their upper arm tight against their trunk and with their forearm at a right angle to the upper arm. A single measurement was taken for both the dominant and non-dominant hands, starting with the non-dominant hand, with values recorded in kilograms (kg) to the nearest integer. The greatest measurement recorded between either the dominant or non-dominant hand was used in the present analysis. In addition, a dichotomous variable pertaining to low HGS was calculated using the cut-offs for low muscle strength of HGS < 27 kg for males and HGS < 16 kg for females, provided by the European Working Group on Sarcopenia in Older People 2 (EWGSOP2) [2].

##### Mobility

The Timed Up-and-Go (TUG) test was used to assess functional mobility and fall risk. The test measures the time taken by the participant to stand up from a standard armchair, walk three meters at their usual pace, turn, walk back to the chair and sit down again [26].

##### Functional limitations

Functional limitations were assessed using self-reported measures of activities of daily living (ADLs), which include basic self-care activities such as dressing, bathing, eating, using the toilet, and getting out of bed [27].

##### Frailty

A frailty index (FI), described in detail elsewhere [28], was derived from 30 self-reported impairments and illnesses collected via the CAPI, encompassing cardiovascular and other chronic conditions, physical and functional limitations, mental health, memory, daytime sleepiness, urinary continence, pain, and polypharmacy. A continuous FI score ranging from 0 to 1 was calculated for participants with complete data by dividing the number of deficits present by the total number assessed. This score was used to categorise individuals as robust (< 0.10), pre-frail (0.10–0.24), or frail (≥ 0.25), based on previously established cut-offs [29], while a dichotomous outcome was also generated pertaining to participants being defined as robust (< 0.10) versus any frailty (≥ 0.10).

#### Covariates

A selection of covariates was incorporated in the multivariate models including age and sex (male, female), education level (3 categories: Primary, Secondary, Higher), alcohol consumption (4 categories: None, Low, Medium, High, based on reported alcohol intake in the FFQ), energy intake (kcal/day) and smoking habits (3 categories, Never, Former, Current). Physical activity was assessed using the International Physical Activity Questionnaire Short Form (IPAQ-SF). Time spent per week in walking, moderate, and vigorous activity was converted to metabolic equivalent (MET)-minutes/week using standard intensity factors (3.3, 4.0, and 8.0 METs, respectively). Total physical activity was calculated as the sum of these components and was included as quintiles of total MET-minutes/week [30]. Area deprivation was assessed using the Northern Ireland Multiple Deprivation Measure (NIMDM), an area-based composite index produced by the Northern Ireland Statistics and Research Agency that ranks small geographic areas in Northern Ireland according to relative deprivation across multiple domains [31]. Household income was segregated into 5 categories based on reported annual household income. Body mass index (BMI) (kg/m^2^) was calculated as weight/height^2^, with participants segregated across four categories: underweight (BMI < 18.5 kg/m^2^), normal weight (BMI 18.5–24.9 kg/m^2^), overweight (BMI 25.0–29.9 kg/m^2^), or obese (BMI ≥ 30.0 kg/m^2^) [32].

### Parallel analysis in TILDA

#### Study population

To evaluate the consistency of findings in NICOLA, participants were drawn from The Irish Longitudinal Study on Ageing (TILDA), a large prospective cohort study of the social, economic and health circumstances of community-dwelling people aged ≥ 50 years in Ireland [19, 21, 33]. The present analysis used data from Wave 3, which was collected between March 2014 and October 2015, including participants with available dietary data, physical function measures, and covariates of interest [34]. Individuals with missing dietary data for the fermented dairy exposure were excluded, resulting in 3999 participants being included in the analysis.

#### Dietary intake and exposure definition

Dietary intake in TILDA was assessed using a semi-quantitative FFQ completed as part of the Wave 3 self-completion questionnaire. The 52-item FFQ was adapted from questionnaires used in the Survey of Lifestyle, Attitudes and Nutrition (SLÁN) 2007 and the EPIC study [34, 35]. Participants reported their habitual frequency of consumption over the preceding year using nine response categories, ranging from “never or rarely” to “six or more times per day”. In TILDA, fermented dairy intake was derived from the available FFQ items for yoghurt and cheese. As the questionnaire did not provide a comprehensive assessment of all fermented dairy products, the resulting combined variable was treated as a proxy measure of the available fermented dairy products available in the TILDA. FFQ responses were converted to approximate daily frequency equivalents (times/day) using standardised mappings. This variable was analysed both as a continuous exposure and as a categorical variable, with participants classified into tertiles as in NICOLA above.

#### Outcome measures and covariates

Outcome measures remained constant across cohorts, pertaining to physical function described above. Detailed methodology relating to the measurements of HGS and TUG specific to TILDA can be found elsewhere [19, 21]. Covariate adjustment in TILDA was aligned closely with NICOLA, the intention was not to create fully harmonised models across cohorts, but rather to evaluate whether broadly comparable patterns of association were observed using the best available cohort-specific variables. In this context, certain variables (including total energy intake, protein intake and area-level deprivation) were not available and therefore not included in TILDA models. Other differences compared to NICOLA included the segregation of smoking status (2 categories: non-smoker, current smoker) and alcohol consumption (2 categories: consumer, abstainer), due to data availability.

### Statistical analysis

For descriptive purposes, the study populations characteristics were summarised across tertiles of fermented dairy intake. P-values for trend were calculated to describe patterns across ordered groups. Associations between fermented dairy intake and physical function outcomes were examined using multivariable regression models, with linear regression implemented for continuous outcomes and logistic regression for binary outcomes. The primary analysis modelled fermented dairy intake as a continuous variable (per 50 g/day increase), with additional models including fermented dairy intake as a categorical exposure (tertiles), with the lowest intake group serving as the reference category. Models were built sequentially: model 0 represented the crude association between the outcome of interest on fermented dairy intake; model 1 adjusted for age and sex; model 2 additionally adjusted for BMI and physical activity; model 3 represented the fully adjusted model, incorporating socioeconomic and lifestyle factors including education, household income, smoking status and alcohol consumption. As indicated above, in NICOLA, models 2 and 3 were additionally adjusted for total energy intake and protein intake, and model 3 for NIMDM. Results from linear models are presented as β coefficients with 95% confidence intervals (CIs), with results from logistic models presented as odds ratios (ORs) with 95% CIs. All analyses were conducted separately within each cohort. Primary inference was based on NICOLA, with analyses subsequently repeated in TILDA to assess whether broadly consistent patterns of association were observed. As described above, cohort-specific differences in dietary exposure measurement and covariate availability necessitated differences in model specification. The rationale for the independent analyses was to examine consistency in the overall patterns of association across two population-based cohorts, rather than to estimate a single pooled effect. Findings were therefore interpreted in terms of the consistency of associations and overall patterns of results across cohorts. Statistical significance was defined as *p* value ≤ 0.05. All statistical analyses were performed in R Statistical Program (version 4.2.2 and 4.5.0).

## Results

### Primary analysis in NICOLA

#### Participant characteristics

A total of 2791 NICOLA participants were included in the analysis. Participant characteristics stratified by tertiles of fermented dairy intake are presented in Table 1.

**Table 1:** Participant characteristics by fermented dairy intake tertiles in NICOLA

Characteristic	Overall N = 2791^1^	Low N = 931^1^	Medium N = 930^1^	High N = 930^1^	*p*-value^2^
Age, years	64 (9)	65 (9)	64 (9)	64 (9)	0.30
Sex, n (%)					< 0.001
Male	1330 (48%)	506 (54%)	460 (49%)	364 (39%)	
Female	1461 (52%)	425 (46%)	470 (51%)	566 (61%)	
BMI kg/m^2^	31.2 (48.9)	31.0 (51.1)	33.0 (63.9)	29.6 (32.3)	0.20
HGS, kg	33 (12)	34 (12)	33 (12)	32 (12)	0.002
Low HGS, n (%)	163 (5.8%)	61 (6.6%)	51 (5.5%)	51 (5.5%)	0.50
Frailty status, n (%)					0.038
Robust	1106 (40%)	351 (38%)	369 (39%)	391 (42%)	
Pre-frail	1022 (37%)	330 (35%)	342 (37%)	350 (38%)	
Frail	658 (23%)	250 (27%)	219 (24%)	189 (20%)	
TUG, seconds	16 (22)	16 (22)	16 (22)	15 (21)	0.010
Prescence of any ADL limitation, n (%)	321 (12%)	110 (11.8%)	121 (13%)	90 (9.7%)	0.037
Fermented dairy intake, g/day	54 (65)	7 (5)	33 (14)	123 (72)	< 0.001
Energy intake, kcal/day	1997 (608)	1802 (582)	2003 (582)	2186 (591)	< 0.001
Protein intake, g/day	83 (25)	75 (24)	83 (24)	91 (25)	< 0.001
Physical activity, weekly METs	23,289 (403,744)	17,214 (271,829)	21,853 (513,908)	30,271 (408,671)	< 0.001
Education level, n (%)					< 0.001
Primary	334 (12%)	145 (16%)	111 (12%)	103 (11%)	
Secondary	1736 (64%)	598 (64%)	589 (63%)	568 (61%)	
Higher	661 (24%)	188 (20%)	230 (25%)	259 (28%)	
Household income, n (%)					
Q1	616 (22%)	84 (9%)	242 (26%)	193 (21%)	
Q2	625 (22%)	401 (43%)	140 (15%)	231 (24%)	
Q3	593 (21%)	130 (14%)	233 (25%)	166 (18%)	
Q4	868 (31%)	307 (33%)	279 (30%)	306 (33%)	
Q5	89 (4%)	9 (1%)	36 (4%)	34 (4%)	
NIMDM, n (%)					0.12
Q1	559 (20%)	171 (18%)	186 (20%)	202 (22%)	
Q2	558 (20%)	174 (19%)	194 (21%)	190 (20%)	
Q3	558 (20%)	177 (19%)	182 (20%)	199 (21%)	
Q4	558 (20%)	196 (21%)	187 (20%)	175 (19%)	
Q5	558 (20%)	213 (23%)	181 (19%)	164 (18%)	
Smoking status, n (%)					0.50
Never	1508 (54%)	473 (51%)	500 (54%)	535 (57%)	
Former	1027 (37%)	350 (37%)	331 (35%)	346 (37%)	
Current	256 (9%)	108 (12%)	99 (11%)	49 (5%)	
Alcohol consumption, n (%)					0.064
None	673 (28%)	237 (25%)	219 (24%)	217 (23%)	
Low	1357 (57%)	596 (64%)	584 (63%)	590 (64%)	
Medium	201 (8.5%)	53 (6%)	78 (8%)	72 (8%)	
High	145 (6.1%)	45 (5%)	49 (5%)	51 (5%)	

^1^Mean (SD); n (%)

^2^*P*-values are presented for descriptive purposes only and do not imply causal inference. General linear model for continuous variables; Chi-squared test for categorical

NICOLA, The Northern Ireland Cohort for the Longitudinal Study of Ageing; BMI, body mass index; MET, metabolic equivalent of task; HGS, hand grip strength; TUG, Timed Up and Go; ADL, activities of daily living

Participant age was similar across fermented dairy intake, with more females in the highest tertile and more males not consuming any fermented dairy foods. Socioeconomic profiles were broadly similar across intake categories, with higher education level amongst participants consuming more fermented dairy foods. With respect to physical function, metrics such as HGS and TUG were similar across categories, although individuals with higher fermented dairy intake were less likely to be frail according to the FI. Higher fermented dairy consumers also had higher total energy and protein intake and were more physically active. Overall, differences across fermented dairy intake groups were present but modest in magnitude.

#### Associations between fermented dairy intake and physical function

**Fig. 2:**
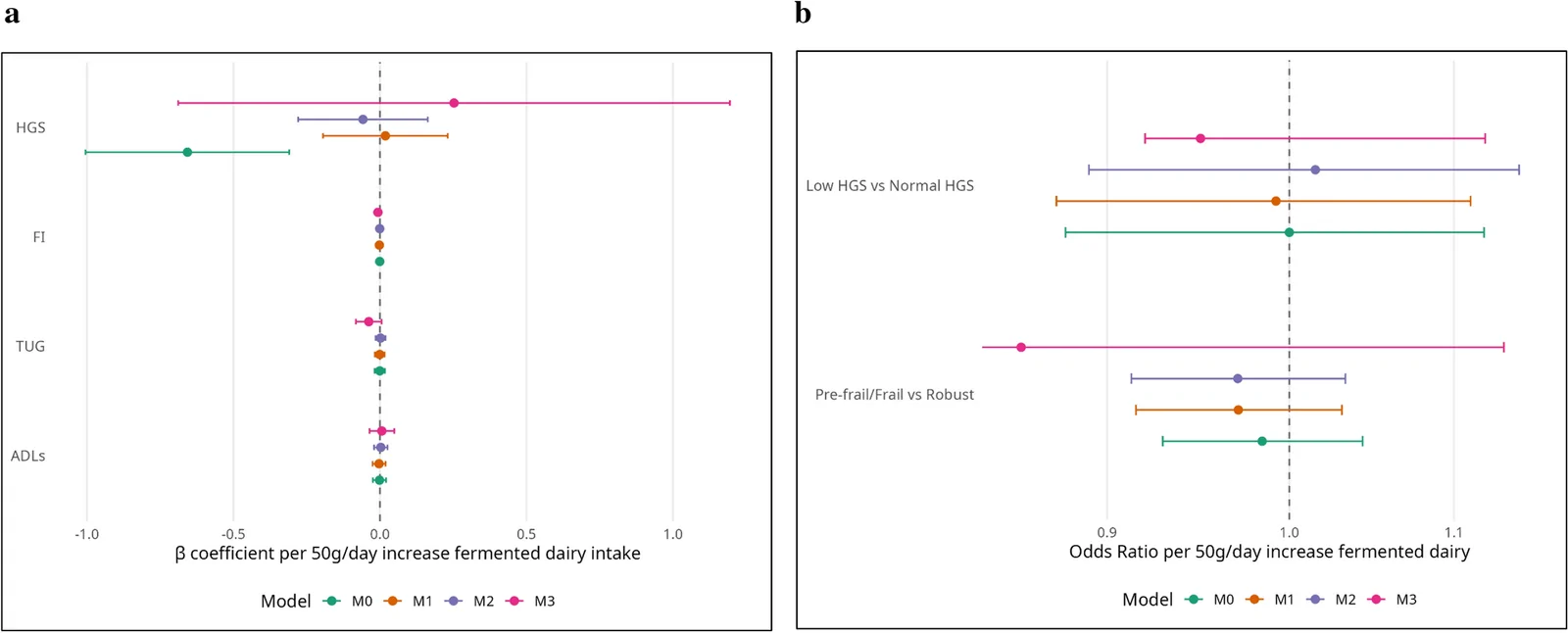
Forest plot depicting the association between fermented dairy intake and physical function in NICOLA. **A** Continuous outcomes modelled with multivariate linear regression. **B** Dichotomous outcomes modelled with multivariate logistic regression. NICOLA, The Northern Ireland Cohort for the Longitudinal Study of Ageing; BMI, HGS, hand grip strength; FI, frailty index; TUG, Timed Up and Go; ADL, activities of daily living

In linear regression models, fermented dairy intake was not significantly associated with HGS and TUG. In unadjusted models, there was a significant inverse association with HGS (β = − 0.66; 95% CI: − 1.00, − 0.31, *p* < 0.001) which was attenuated after adjustment (β = 0.25; 95% CI: − 0.69, 1.19, *p* = 0.597). There were also no associations identified between fermented dairy intake and FI and ADL score (Table 2, Fig. 2A). This was also the case when modelling dichotomous outcomes, with no associations between fermented dairy intake and low HGS or frailty status (Table 3, Fig. 2B).

**Table 2:** Association between fermented dairy intake and physical function outcomes in NICOLA and TILDA

Outcome and Model	β (95% CI)	P value
NICOLA		
*HGS*		
Model 0	− 0.66 (− 1.00, − 0.31)	< 0.001
Model 1	0.02 (− 0.19, 0.23)	0.865
Model 2	− 0.06 (− 0.28, 0.16)	0.606
Model 3	0.25 (− 0.69, 1.19)	0.597
*FI*		
Model 0	− 0.0001 (− 0.005, 0.002)	0.630
Model 1	− 0.002(− 0.006, 0.002)	0.247
Model 2	− 0.0009 (− 0.005, 0.003)	0.644
Model 3	− 0.007 (− 0.018, 0.004)	0.220
*TUG*		
Model 0	− 0.0007 (− 0.018, 0.017)	0.940
Model 1	− 0.0009 (− 0.017, 0.015)	0.916
Model 2	0.0019 (− 0.015, 0.019)	0.828
Model 3	− 0.04 (− 0.082, 0.005)	0.086
*ADLs*		
Model 0	− 0.002 (− 0.024, 0.021)	0.884
Model 1	− 0.003 (− 0.025, 0.019)	0.772
Model 2	0.003 (− 0.02, 0.03)	0.814
Model 3	0.006 (− 0.036, 0.048)	0.769
TILDA		
*HGS*		
Model 0	− 0.86 (−1.19, − 0.52)	< 0.001
Model 1	− 0.05 (− 0.26, 0.17)	0.658
Model 2	− 0.10 (− 0.78, 0.59)	0.784
Model 3	− 0.05 (− 0.82, 0.73)	0.907
*FI*		
Model 0	− 0.01 (− 0.03, 0.02)	0.665
Model 1	− 0.01 (− 0.03, 0.01)	0.453
Model 2	0.00 (− 0.05, 0.05)	0.973
Model 3	0.01 (− 0.04, 0.07)	0.613
*TUG*		
Model 0	− 0.01 (− 0.02, 0.00)	0.029
Model 1	− 0.01 (− 0.02, 0.00)	0.058
Model 2	− 0.01 (− 0.03, 0.01)	0.308
Model 3	0.00 (− 0.02, 0.02)	0.996
*ADLs*		
Model 0	− 0.01 (− 0.02, 0.01)	0.431
Model 1	− 0.01 (− 0.02, 0.01)	0.418
Model 2	0.00 (− 0.03, 0.03)	0.843
Model 3	0.00 (− 0.02, 0.02)	0.838

Estimates derived from multivariable linear regression models. For NICOLA, fermented dairy intake was modelled as a continuous variable per 50 g/day increase. Model 0 represents the crude association between fermented dairy intake and the outcome. Model 1 adjusts for age and sex. Model 2 additionally adjusts for body mass index (BMI), energy intake, protein intake and physical activity. Model 3 represents the fully adjusted model, additionally including socioeconomic and lifestyle factors (education, household income, area-level deprivation, smoking status, and alcohol consumption). For TILDA, fermented dairy intake was modelled as daily frequency calculated from FFQ, models were adjusted with the same covariates as NICOLA, with the exceptions of Model 2 (energy and protein intake omission) and Model 3 (area-level deprivation omission). Abbreviations: NICOLA, The Northern Ireland Cohort for the Longitudinal Study of Ageing; TILDA, The Irish Longitudinal Study on Ageing; HGS, hand grip strength; FI, frailty index; TUG, Timed Up and Go; ADLs, activities of daily living; CI, confidence interval

**Table 3:** Association between fermented dairy intake and dichotomous physical function outcomes in NICOLA and TILDA

Outcome / Model	OR (95% CI)	*P* value
NICOLA		
*Low HGS versus Normal HGS*		
Model 0	1.00 (0.88, 1.12)	1.000
Model 1	0.99 (0.87, 1.11)	0.899
Model 2	1.02 (0.89, 1.14)	0.811
Model 3	0.95 (0.92, 1.12)	1.000
*Frail/pre-frail versus Robust*		
Model 0	0.98 (0.93, 1.04)	0.594
Model 1	0.97 (0.91, 1.03)	0.331
Model 2	0.97 (0.91, 1.03)	0.344
Model 3	0.86 (0.64, 1.13)	0.282
TILDA		
*Low HGS versus Normal HGS*		
Model 0	1.00 (0.90, 1.11)	0.941
Model 1	1.02 (0.92, 1.13)	0.695
Model 2	1.31 (0.85, 1.92)	0.186
Model 3	1.51 (0.97, 2.30)	0.056
*Frail/pre-frail versus Robust*		
Model 0	0.99 (0.93, 1.07)	0.876
Model 1	0.99 (0.91, 1.06)	0.716
Model 2	1.03 (0.79, 1.34)	0.805
Model 3	1.16 (0.84, 1.58)	0.342

Estimates derived from multivariable logistic regression models. For NICOLA, fermented dairy intake was modelled as a continuous variable per 50 g/day increase. Model 0 represents the crude association between fermented dairy intake and the outcome. Model 1 adjusts for age and sex. Model 2 additionally adjusts for body mass index (BMI), energy intake, protein intake and physical activity. Model 3 represents the fully adjusted model, additionally including socioeconomic and lifestyle factors (education, household income, area-level deprivation, smoking status, and alcohol consumption). For TILDA, fermented dairy intake was modelled as daily frequency calculated from FFQ, models were adjusted with the same covariates as NICOLA, with the exceptions of Model 2 (energy and protein intake omission) and Model 3 (area-level deprivation omission). Abbreviations: NICOLA, The Northern Ireland Cohort for the Longitudinal Study of Ageing; TILDA, The Irish Longitudinal Study on Ageing; HGS, hand grip strength; OR, odds ratio; CI, confidence interval

When analysed by tertiles, there were significant associations in unadjusted models between participants in the highest category of fermented dairy intake and lower HGS (Model 0, β = − 1.93; 95% CI: − 3.10, − 0.75, *p* = 0.001) and FI (Model 1, β = − 0.02; 95% CI: − 0.03, 0.00, *p* = 0.014). However, these associations were attenuated and inconsistent after multivariable adjustment (HGS, β = 1.64; 95% CI: − 1.67, 4.94, *p* = 0.329; FI, β = − 0.01; 95% CI: − 0.05, 0.03, *p* = 0.729), and no clear dose–response relationship was observed (Table S2). In less adjusted models (Model 0–2), tertile based analyses indicated an increased odds of being considered pre-frail or frail according to the FI, however this association was attenuated in the fully adjusted model (OR = 0.30; 95% CI: 0.07–1.13; *p* = 0.082). There was no association for odds of low HGS (Table S4).

### Replication analysis in TILDA

#### Participant characteristics

To assess the consistency of findings, analyses were repeated in an independent cohort of 3999 TILDA participants. Participant characteristics stratified by fermented dairy intake are presented in Table S1. Similar to NICOLA, higher fermented dairy intake was associated with differences in demographic and socioeconomic characteristics, including a higher proportion of females and more favourable education and wealth profiles. Patterns for physical function outcomes were less consistent across intake groups, including HGS, TUG and frailty status.

#### Associations between fermented dairy intake and physical function

**Fig. 3:**
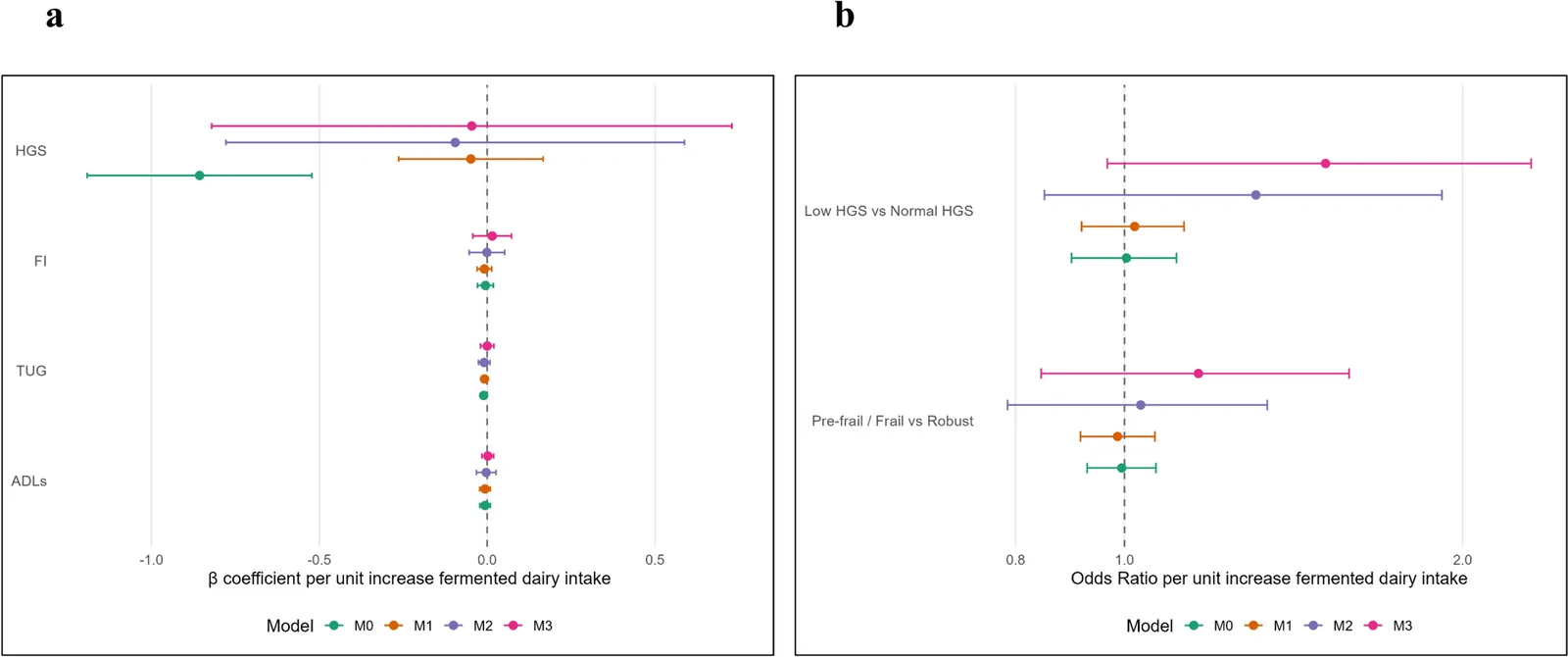
Forest plot depicting the association between fermented dairy intake and physical function in TILDA. **A** Continuous outcomes modelled with multivariate linear regression. **B** Dichotomous outcomes modelled with multivariate logistic regression. TILDA, The Irish Longitudinal Study on Ageing; BMI, HGS, hand grip strength; FI, frailty index; TUG, Timed Up and Go; ADL, activities of daily living

In unadjusted models, higher fermented dairy intake was associated with lower HGS and modestly faster TUG times. However, these associations were attenuated after adjustment in fully adjusted models (HGS (β = − 0.05; 95% CI: − 0.82, 0.73; *p* = 0.907), TUG (β = 0.00; 95% CI: − 0.02, 0.02; *p* = 0.996)) (Table 2**, **Fig. 3A). Similarly, no consistent associations were observed for binary outcomes, low HGS and frailty (Table 3**, **Fig. 3B).

In tertile-based analyses, initial unadjusted models indicated differences in HGS and TUG across levels of fermented dairy intake; however, these were not maintained after multivariable adjustment (Table S3). In fully adjusted models, there was evidence of higher odds of low HGS among individuals in the highest intake tertile compared with the lowest (OR = 2.71; 95% CI: 1.12, 6.88; *p* = 0.030), although this association was not apparent when modelling HGS as a continuous outcome (β = 0.09; 95% CI: −1.34, 1.52; *p* = 0.901). There were no consistent findings for associations between fermented dairy intake and frailty (Table S5).

To understand what may be driving the association between fermented dairy intake and higher odds of low HGS in TILDA, yoghurt and cheese intake were examined separately to explore potential heterogeneity in associations across fermented dairy subtypes. In these analyses, yoghurt intake appeared to drive associations observed in unadjusted models, particularly for HGS (β = −1.52; 95% CI: −1.99, −1.06; *p* < 0.001). However, these associations were attenuated after adjustment for confounding factors (β = 0.30; 95% CI: −0.90, 1.51; *p* = 0.619) (Figure S1). No consistent associations were observed for cheese intake across outcomes (Table S6).

## Discussion

To our knowledge, this is the first study to examine fermented dairy intake in relation to multiple domains of physical function across two complementary population-based cohorts in Ireland. Previous epidemiological studies have typically focused on total dairy or overall protein intake in relation to frailty, sarcopenia, and physical performance in older adults [36–39], with relatively few examining fermented dairy subtypes [40, 41], despite their potential musculoskeletal benefits.

Across the two cohorts, fermented dairy intake was not independently associated with objective or self-reported measures of physical function after adjustment for demographic, lifestyle, and socioeconomic factors. Although crude analyses suggested associations between higher fermented dairy intake and functional outcomes, these findings were inconsistent across endpoints and were attenuated in fully adjusted models.

In NICOLA and TILDA, initial unadjusted associations between fermented dairy intake and physical function were largely attributable to confounding factors. In NICOLA, modest associations with HGS and FI did not persist after multivariable adjustment, and no clear dose–response relationship was evident across intake tertiles. Similarly, in TILDA, unadjusted associations were fully attenuated after adjustment, with no consistent relationships observed across continuous or categorical outcomes. Although a single association was identified between higher fermented dairy intake and increased odds of low HGS in TILDA, this was not supported by continuous analyses or by other functional measures, suggesting a likely spurious finding.

The present findings are broadly consistent with the limited existing observational literature, which has generally reported weak or inconsistent associations between dairy or fermented dairy consumption and physical function after adjustment for confounding factors [40, 41]. Where associations have been identified, they are typically small in magnitude and inconsistent across functional domains and study populations. The current analysis therefore adds to the growing body of evidence suggesting that findings from minimally adjusted models may partly reflect broader differences in health behaviours, diet quality, and socioeconomic status, rather than an independent biological effect of fermented dairy foods.

Reverse causation is also a plausible explanation for the observed patterns. Older adults experiencing declining physical function may preferentially consume fermented dairy foods such as yoghurt or cottage cheese due to ease of consumption and palatability, or following dietary advice aimed at maintaining energy and protein intake in the context of declining health [42]. In cross-sectional analyses, this may generate spurious positive associations between fermented dairy intake and poorer physical function, where the direction of effect is reversed or bidirectional [40, 41]. Subtype analyses in TILDA indicated that yoghurt intake may have driven associations between fermented dairy intake and low HGS; however, the attenuation of associations after adjustment, together with the absence of consistent findings for cheese, does not support a specific beneficial or harmful effect of fermented dairy subtypes.

The inconsistent and attenuated associations observed across analyses highlight the need for robust randomised controlled trials (RCTs) [43] to clarify the role of fermented dairy foods in musculoskeletal health across both healthy and ageing populations. While observational data can identify population-level patterns, they remain limited by confounding and potential for reverse causation, particularly in older adults, where dietary behaviours may change in response to declining health status. In addition, the effects of dietary protein and fermented dairy foods on skeletal muscle are likely to depend on concurrent physical activity, particularly resistance exercise. While fermented dairy foods may provide nutrients and bioactive compounds that support muscle health, muscle maintenance and adaptation are strongly influenced by mechanical loading [6], and nutritional benefits may be limited in the absence of an adequate exercise stimulus. Future studies should therefore consider the interactive effects of fermented dairy intake and physical activity, particularly resistance training, when evaluating associations with muscle strength and physical function in older adults. RCTs are therefore needed to determine whether fermented dairy foods exert a causal effect on skeletal muscle outcomes, including strength, quality, function, as well as outcomes with greater mechanistic granularity, such as muscle protein synthesis and anabolic responses.

From a mechanistic perspective, this work was motivated by the hypothesised role of fermented foods in modulating the gut-muscle axis. Fermented dairy foods contain high-quality protein, microbial components, and bioactive compounds, that may plausibly influence skeletal muscle metabolism through effects on gut microbiome composition and function, inflammation, and endocrine signalling [15]. However, a key limitation of the present study is the absence of microbiome or metabolomic data in either cohort at the time of analysis. Consequently, it was not possible to directly evaluate whether fermented dairy intake is associated with gut microbial composition or whether such pathways mediated relationships with physical function. In this context, microbiome data from Wave 3 of NICOLA will provide an important opportunity to revisit these questions using integrated dietary and microbial profiling. The present study was therefore primarily hypothesis-generating and informed by emerging evidence relating to the gut-muscle axis. Although microbiome data were unavailable, the analyses were designed to generate population-level insights and inform future mechanistic and interventional studies.

This study has several strengths. It utilises two large, well-phenotyped population-based cohorts with complementary designs and comparable analytical approaches, allowing replication of findings across independent datasets. A broad range of objective and self-reported measures of physical function were included, alongside comprehensive adjustment for demographic, socioeconomic, lifestyle, and health-related confounders. The use of both continuous and categorical exposure models further strengthens the robustness of the findings. The novelty of the work is an additional strength, providing a foundation for future research investigating the role of fermented dairy foods in healthy skeletal muscle ageing and the potential contribution of the gut-muscle axis.

However, several limitations should be acknowledged. The cross-sectional design precludes inference regarding temporality and increases susceptibility to reverse causation, particularly for outcomes related to physical function and frailty, where declining health status may influence dietary behaviours [44, 45]. Dietary assessment was conducted using FFQs, which are subject to measurement error and recall bias [46, 47]. Residual confounding cannot be excluded, particularly relating to unmeasured dietary patterns, health behaviours, and disease status. Finally, some subgroup analyses were limited by small numbers within exposure categories, potentially reducing statistical power to detect modest associations.

Another important limitation relates to exposure measurement. Neither cohort included a dedicated instrument specifically designed to capture fermented food intake in detail. As a result, classification of fermented dairy intake was based on available dietary assessment tools, which may not fully capture variation in fermentation type, processing, microbial content, or frequency of intake. Recent advances in the development of fermented food-specific FFQs may improve exposure characterisation in future studies and allow for more precise investigation of associations between fermented foods and health outcomes [48]. In addition, the exposure definitions for fermented dairy intake were not fully harmonised across cohorts. In NICOLA, intake was quantified in grams per day using a more granular FFQ-based measure, whereas in TILDA, a proxy measure based on the reported frequency of yoghurt and cheese consumption was used. These measures therefore capture related, but not identical, aspects of fermented dairy consumption and may differ in their susceptibility to exposure misclassification. Consequently, direct comparisons of effect estimates between cohorts should be made cautiously. Nevertheless, the use of conceptually aligned exposures enabled the examination of associations within two large, independent population-based cohorts using the dietary data available in each study. The present study was not designed as a formally harmonised cohort analysis; therefore, findings should be interpreted in terms of the consistency of associations and overall patterns of results rather than the direct equivalence of exposure measures or effect estimates.

In conclusion, across two complementary population-based cohorts on the island of Ireland, fermented dairy intake was not independently associated with physical function or frailty after adjustment for key confounders. While crude associations were observed, these were not robust and were largely explained by demographic, socioeconomic, and lifestyle factors. Taken together, these findings underscore the importance of integrative, mechanistically informed longitudinal and interventional studies to establish whether fermented dairy foods play a meaningful role in mitigating age-related declines in physical function and to further elucidate the contribution of the gut-muscle axis in this context.

## Supplementary Information

Below is the link to the electronic supplementary material.

Supplementary file1 (DOCX 128 kb)

## Data Availability

The data included are available from the NICOLA Data Access Committee upon request (https://www.qub.ac.uk/sites/NICOLA/UseOurData/). Further to this, the data and/or meta-data from the health assessment (along with the CAPI and SCQ data) is deposited in various data repositories including the Dementias Platform UK (DPUK), UK Data Service (UKDS), UK Longitudinal Linkage Collaboration (UK LLC) and Gateway to Global Ageing (G2G) which hold similar data from other cohort studies; this helps to maximise data sharing and foster research collaborations. TILDA data can be accessed via the Irish Social Science Data Archive (www.ucd.ie/issda). The publicly accessible dataset files are hosted by the Irish Social Science Data Archive based in University College Dublin, and the Interuniversity Consortium for Political and Social Research. Researchers wishing to access the data must complete a request form, available on the ISSDA website. The data that have been used is confidential.
