# Supplementary material for: Fermented dairy intake and markers of musculoskeletal ageing on the island of Ireland: insights from the Northern Ireland Cohort for the Longitudinal Study of Ageing (NICOLA) and The Irish Longitudinal Study on Ageing (TILDA)
Source: Eur J Nutr. 2026 Nov 4;65(8):295. doi: 10.1007/s00394-026-04150-0 (PMC13736369; doi:10.1007/s00394-026-04150-0)
Supplement: Supplementary file 1 [file 394_2026_4150_MOESM1_ESM.docx]

**Fermented dairy intake and markers of musculoskeletal ageing on the island of Ireland: insights from the Northern Ireland Cohort for the Longitudinal Study of Ageing (NICOLA) and The Irish Longitudinal Study on Ageing (TILDA)**

**Supplementary material**

| **Table S1.** Participant characteristics by fermented dairy intake tertiles in TILDA | **2** |
| --- | --- |
| **Table S2.** Association between fermented dairy intake tertiles and continuous physical function outcomes in NICOLA | **4** |
| **Table S3.** Association between fermented dairy intake tertiles and continuous physical function outcomes in TILDA | **6** |
| **Table S4.** Association between fermented dairy intake tertiles and dichotomous physical function outcomes in NICOLA | **8** |
| **Table S5.** Association between fermented dairy intake tertiles and dichotomous physical function outcomes in TILDA | **9** |
| **Table S6**. Associations between decomposed fermented dairy variable components and physical function outcomes in TILDA | **10** |
| **Figure S1.** Forest plot depicting association between yoghurt and cheese consumption with physical function in TILDA. | **13** |

| **Table S1. Participant characteristics by fermented dairy intake tertiles in TILDA** | | | | | |
| --- | --- | --- | --- | --- | --- |
| **Characteristic** | **Overall**  N = 3,999^1^ | **Low**  N = 1,475^1^ | **Medium**  N = 1,264^1^ | **High**  N = 1,260^1^ | **p-value**^2^ |
| **Age, years** | 66 (9) | 66 (9) | 65 (9) | 65 (8) | 0.002 |
| **Sex, n (%)** |  |  |  |  | <0.001 |
| Male | 1,824 (46%) | 765 (52%) | 607 (48%) | 452 (36%) |  |
| Female | 2,175 (54%) | 710 (48%) | 657 (52%) | 808 (64%) |  |
| **BMI, kg/m²** | 28.6 (5.1) | 28.7 (5.4) | 28.5 (5.0) | 28.4 (4.9) | 0.30 |
| **HGS, kg** | 28 (10) | 29 (10) | 29 (9) | 27 (9) | <0.001 |
| **Low HGS, n (%)** | 473 (12%) | 202 (13.7%) | 125 (9.9%) | 146 (12%) | 0.017 |
| **Frailty status, n (%)** |  |  |  |  | 0.005 |
| Robust | 2,253 (56%) | 780 (53%) | 755 (60%) | 718 (57%) |  |
| Pre-frail | 1,324 (33%) | 516 (35%) | 392 (31%) | 416 (33%) |  |
| Frail | 422 (11%) | 179 (12%) | 117 (9.3%) | 126 (10%) |  |
| **TUG, seconds** | 9.68 (4.09) | 10.17 (4.6) | 9.48 (3.62) | 9.53 (4.01) | <0.001 |
| **Prescence of any ADL limitation, n (%)** | 167 (4.2%) | 72 (4.9%) | 54 (4.3%) | 41 (3.3%) | 0.076 |
| **Physical activity, weekly METs** | 6,237 (4,210) | 6,321 (4,300) | 6,154 (3,916) | 6,374 (4,569) | 0.90 |
| **Education level, n (%)** |  |  |  |  | <0.001 |
| Primary | 813 (20%) | 367 (25%) | 229 (18%) | 217 (17%) |  |
| Secondary | 1,597 (40%) | 597 (40%) | 509 (40%) | 491 (39%) |  |
| Higher | 1,588 (40%) | 511 (35%) | 526 (42%) | 551 (44%) |  |
| **Household income, n (%)** |  |  |  |  | <0.001 |
| Q1 | 507 (17%) | 308 (21%) | 202 (16%) | 164 (13%) |  |
| Q2 | 581 (19%) | 310 (21%) | 215 (17%) | 214 (17%) |  |
| Q3 | 595 (20%) | 280 (19%) | 253 (20%) | 252 (20%) |  |
| Q4 | 668 (22%) | 282 (19%) | 291 (23%) | 328 (26%) |  |
| Q5 | 678 (22%) | 295 (20%) | 303 (24%) | 302 (24%) |  |
| **Smoking status, n (%)** |  |  |  |  | <0.001 |
| Non-smoker | 3,577 (89%) | 1,277 (87%) | 1,154 (91%) | 1,146 (91%) |  |
| Current smoker | 421 (11%) | 198 (13%) | 109 (8.6%) | 114 (9.0%) |  |
| **Alcohol consumer, n (%)** | 3,228 (93%) | 1,63 (94%) | 1,041 (94%) | 1,024 (92%) | 0.005 |
| ^1^Mean (SD); n (%) | | | | | |
| ^2^P values for descriptive purposes only. Generated via Kruskal-Wallis rank sum test for continuous outcomes and Pearson's Chi-squared test for categorical  TILDA, The Irish Longitudinal Study on Ageing; BMI, body mass index; MET, metabolic equivalent of task; HGS, hand grip strength; TUG, Timed Up and Go; ADL, activities of daily living. | | | | | |

| **Table S2. Association between fermented dairy intake tertiles and continuous physical function outcomes in NICOLA** | | |
| --- | --- | --- |
| **Outcome / Comparison / Model** | **β (95% CI)** | **p-value** |
| **HGS** |  |  |
| Medium vs Low – Model 0 | -0.55 (-1.72, 0.62) | 0.359 |
| Medium vs Low – Model 1 | -0.12 (-0.80, 0.60) | 0.743 |
| Medium vs Low – Model 2 | -0.26 (-0.98, 0.46) | 0.479 |
| Medium vs Low – Model 3 | 0.98 (-2.23, 4.18) | 0.548 |
| High vs Low – Model 0 | -1.93 (-3.10, -0.75) | 0.001 |
| High vs Low – Model 1 | 0.04 (-0.68, 0.76) | 0.905 |
| High vs Low – Model 2 | -0.18 (-0.930, 0.56) | 0.629 |
| High vs Low – Model 3 | 1.64 (-1.67, 4.94) | 0.329 |
| **FI** |  |  |
| Medium vs Low – Model 0 | 0.000 (-0.010, 0.010) | 0.903 |
| Medium vs Low – Model 1 | 0.000 (-0.010, 0.010) | 0.897 |
| Medium vs Low – Model 2 | 0.000 (-0.010, 0.010) | 0.967 |
| Medium vs Low – Model 3 | 0.010 (-0.030, 0.040) | 0.752 |
| High vs Low – Model 0 | -0.010 (-0.030, 0.000) | 0.038 |
| High vs Low – Model 1 | -0.020 (-0.030, 0.000) | 0.014 |
| High vs Low – Model 2 | -0.010 (-0.030, 0.000) | 0.066 |
| High vs Low – Model 3 | -0.010 (-0.050, 0.030) | 0.729 |
| **TUG** |  |  |
| Medium vs Low – Model 0 | 0.000 (-0.060, 0.060) | 0.903 |
| Medium vs Low – Model 1 | 0.020 (-0.040, 0.070) | 0.579 |
| Medium vs Low – Model 2 | 0.020 (-0.040, 0.070) | 0.595 |
| Medium vs Low – Model 3 | 0.100 (-0.040, 0.240) | 0.174 |
| High vs Low – Model 0 | -0.040 (-0.100, 0.020) | 0.205 |
| High vs Low – Model 1 | -0.020 (-0.070, 0.040) | 0.523 |
| High vs Low – Model 2 | -0.010 (-0.070, 0.050) | 0.695 |
| High vs Low – Model 3 | -0.020 (-0.170, 0.130) | 0.758 |
| **ADLs** |  |  |
| Medium vs Low – Model 0 | 0.040 (-0.030, 0.120) | 0.258 |
| Medium vs Low – Model 1 | 0.040 (-0.030, 0.120) | 0.277 |
| Medium vs Low – Model 2 | 0.050 (-0.030, 0.120) | 0.244 |
| Medium vs Low – Model 3 | 0.070 (-0.070, 0.210) | 0.311 |
| High vs Low – Model 0 | -0.020 (-0.100, 0.050) | 0.523 |
| High vs Low – Model 1 | -0.030 (-0.100, 0.050) | 0.493 |
| High vs Low – Model 2 | -0.010 (-0.090, 0.070) | 0.825 |
| High vs Low – Model 3 | 0.050 (-0.090, 0.200) | 0.461 |
| Estimates derived from multivariable linear regression models. Model 0 represents the crude association between fermented dairy intake and the outcome. Model 1 adjusts for age and sex. Model 2 additionally adjusts for body mass index (BMI), energy intake, protein intake and physical activity. Model 3 represents the fully adjusted model, additionally including socioeconomic and lifestyle factors (education, household income, area-level deprivation, smoking status, and alcohol consumption). Abbreviations: NICOLA, The Northern Ireland Cohort for the Longitudinal Study of Ageing; HGS, hand grip strength; FI, frailty index; TUG, Timed Up and Go; ADL, activities of daily living; CI, confidence interval. | | |

| **Table S3. Association between fermented dairy intake tertiles and continuous physical function outcomes in TILDA** | | |
| --- | --- | --- |
| **Outcome / Comparison / Model** | **β (95% CI)** | **p-value** |
| **HGS** |  |  |
| Medium vs Low – Model 0 | −0.06 (−0.78, 0.66) | 0.862 |
| Medium vs Low – Model 1 | 0.16 (−0.30, 0.62) | 0.494 |
| Medium vs Low – Model 2 | 0.13 (−1.08, 1.33) | 0.837 |
| Medium vs Low – Model 3 | 0.25 (−1.18, 1.69) | 0.729 |
| High vs Low – Model 0 | −1.89 (−2.61, −1.17) | <0.001 |
| High vs Low – Model 1 | 0.12 (−0.34, 0.59) | 0.601 |
| High vs Low – Model 2 | −0.12 (−1.34, 1.09) | 0.844 |
| High vs Low – Model 3 | 0.09 (−1.34, 1.52) | 0.901 |
| **FI** |  |  |
| Medium vs Low – Model 0 | −0.10 (−0.15, −0.05) | <0.001 |
| Medium vs Low – Model 1 | −0.07 (−0.12, −0.03) | 0.003 |
| Medium vs Low – Model 2 | −0.04 (−0.13, 0.05) | 0.417 |
| Medium vs Low – Model 3 | −0.05 (−0.15, 0.06) | 0.385 |
| High vs Low – Model 0 | −0.06 (−0.12, −0.01) | 0.013 |
| High vs Low – Model 1 | −0.06 (−0.10, −0.01) | 0.022 |
| High vs Low – Model 2 | −0.06 (−0.15, 0.04) | 0.245 |
| High vs Low – Model 3 | −0.04 (−0.15, 0.06) | 0.412 |
| **TUG** |  |  |
| Medium vs Low – Model 0 | −0.04 (−0.06, −0.02) | <0.001 |
| Medium vs Low – Model 1 | −0.02 (−0.04, 0.00) | 0.013 |
| Medium vs Low – Model 2 | −0.02 (−0.05, 0.01) | 0.220 |
| Medium vs Low – Model 3 | −0.01 (−0.05, 0.03) | 0.604 |
| High vs Low – Model 0 | −0.04 (−0.06, −0.02) | <0.001 |
| High vs Low – Model 1 | −0.02 (−0.04, −0.01) | 0.008 |
| High vs Low – Model 2 | −0.01 (−0.04, 0.02) | 0.614 |
| High vs Low – Model 3 | 0.01 (−0.03, 0.04) | 0.698 |
| **ADL Score** |  |  |
| Medium vs Low – Model 0 | −0.01 (−0.04, 0.03) | 0.673 |
| Medium vs Low – Model 1 | 0.00 (−0.04, 0.03) | 0.878 |
| Medium vs Low – Model 2 | 0.03 (−0.02, 0.08) | 0.250 |
| Medium vs Low – Model 3 | 0.00 (−0.03, 0.03) | 0.889 |
| High vs Low – Model 0 | −0.02 (−0.05, 0.02) | 0.282 |
| High vs Low – Model 1 | −0.02 (−0.05, 0.02) | 0.344 |
| High vs Low – Model 2 | 0.01 (−0.05, 0.06) | 0.842 |
| High vs Low – Model 3 | 0.00 (−0.03, 0.04) | 0.798 |
| Estimates derived from multivariable linear regression models. Model 0 represents the crude association between fermented dairy intake (tertiles) and the outcome. Model 1 adjusts for age and sex. Model 2 additionally adjusts for body mass index (BMI) and physical activity. Model 3 represents the fully adjusted model, additionally including socioeconomic and lifestyle factors (education, household income, smoking status, and alcohol consumption). Abbreviations: TILDA, The Irish Longitudinal Study on Ageing; BMI, body mass index; HGS, hand grip strength; FI, frailty index; TUG, Timed Up and Go; ADL, activities of daily living; CI, confidence interval. | | |

| **Table S4. Association between fermented dairy intake tertiles and dichotomous physical function outcomes in NICOLA** | | |
| --- | --- | --- |
| **Outcome / Comparison / Model** | **OR (95% CI)** | **P value** |
| **Low HGS vs Normal HGS** |  |  |
| Medium vs Low – Model 0 | 0.77 (0.52, 1.15) | 0.207 |
| Medium vs Low – Model 1 | 0.83 (0.54, 1.26) | 0.379 |
| Medium vs Low – Model 2 | 1.15 (0.58, 2.52) | 0.702 |
| Medium vs Low – Model 3 | 1.18 (0.60, 2.66) | 0.756 |
| High vs Low – Model 0 | 0.77 (0.52, 1.15) | 0.207 |
| High vs Low – Model 1 | 0.83 (0.54, 1.27) | 0.388 |
| High vs Low – Model 2 | 1.25 (0.62, 2.76) | 0.562 |
| High vs Low – Model 3 | 1.32 (0.67, 3.42) | 0.643 |
| **Frail/pre-frail vs Robust** |  |  |
| Medium vs Low – Model 0 | 0.78 (0.57, 1.08) | 0.136 |
| Medium vs Low – Model 1 | 0.75 (0.54, 1.04) | 0.091 |
| Medium vs Low – Model 2 | 0.75 (0.54, 1.05) | 0.094 |
| Medium vs Low – Model 3 | 0.51 (0.13, 1.91) | 0.322 |
| High vs Low – Model 0 | 0.70 (0.51, 0.97) | 0.032 |
| High vs Low – Model 1 | 0.66 (0.47, 0.92) | 0.014 |
| High vs Low – Model 2 | 0.66 (0.47, 0.92) | 0.016 |
| High vs Low – Model 3 | 0.30 (0.07, 1.13) | 0.082 |
| Estimates derived from multivariable logistic regression models. Model 0 represents the crude association between fermented dairy intake and the outcome. Model 1 adjusts for age and sex. Model 2 additionally adjusts for body mass index (BMI), energy intake, protein intake and physical activity. Model 3 represents the fully adjusted model, additionally including socioeconomic and lifestyle factors (education, household income, area-level deprivation, smoking status, and alcohol consumption). Abbreviations: NICOLA, The Northern Ireland Cohort for the Longitudinal Study of Ageing; HGS, hand grip strength; CI, confidence interval. | | |

| **Table S5. Association between fermented dairy intake tertiles and dichotomous physical function outcomes in TILDA** | | |
| --- | --- | --- |
| **Outcome / Comparison / Model** | **OR (95% CI)** | **P value** |
| **Low HGS vs Normal HGS** |  |  |
| Medium vs Low – Model 0 | 0.68 (0.54, 0.86) | 0.002 |
| Medium vs Low – Model 1 | 0.74 (0.58, 0.95) | 0.019 |
| Medium vs Low – Model 2 | 0.45 (0.14, 1.23) | 0.141 |
| Medium vs Low – Model 3 | 0.59 (0.15, 1.87) | 0.389 |
| High vs Low – Model 0 | 0.83 (0.66, 1.04) | 0.106 |
| High vs Low – Model 1 | 0.93 (0.73, 1.18) | 0.529 |
| High vs Low – Model 2 | 1.96 (0.92, 4.23) | 0.082 |
| High vs Low – Model 3 | 2.71 (1.12, 6.88) | 0.030 |
| **Frail/pre-frail vs Robust** |  |  |
| Medium vs Low – Model 0 | 0.75 (0.64, 0.87) | <0.001 |
| Medium vs Low – Model 1 | 0.78 (0.66, 0.92) | 0.003 |
| Medium vs Low – Model 2 | 0.86 (0.54, 1.36) | 0.521 |
| Medium vs Low – Model 3 | 0.82 (0.46, 1.47) | 0.507 |
| High vs Low – Model 0 | 0.84 (0.72, 0.97) | 0.021 |
| High vs Low – Model 1 | 0.85 (0.72, 1.00) | 0.046 |
| High vs Low – Model 2 | 0.79 (0.49, 1.26) | 0.327 |
| High vs Low – Model 3 | 0.87 (0.48, 1.54) | 0.625 |
| Estimates derived from multivariable logistic regression models. Model 0 represents the crude association between fermented dairy intake (tertiles) and the outcome. Model 1 adjusts for age and sex. Model 2 additionally adjusts for body mass index (BMI) and physical activity. Model 3 represents the fully adjusted model, additionally including socioeconomic and lifestyle factors (education, household income, smoking status, and alcohol consumption). Abbreviations: TILDA, The Irish Longitudinal Study on Ageing; HGS, hand grip strength; TUG, Timed Up and Go; ADL, activities of daily living; CI, confidence interval. | | |

| **Table S6. Associations between decomposed fermented dairy variable components and physical function outcomes in TILDA** | | |
| --- | --- | --- |
| **Outcome / Exposure / Model** | **Estimate / OR (95% CI)** | **p-value** |
| **Continuous outcomes** | | |
| **HGS** |  |  |
| Yoghurt – Model 0 | −1.52 (−1.99, −1.06) | <0.001 |
| Yoghurt – Model 1 | −0.03 (−0.33, 0.27) | 0.833 |
| Yoghurt – Model 2 | 0.05 (−1.00, 1.11) | 0.920 |
| Yoghurt – Model 3 | 0.30 (−0.90, 1.51) | 0.619 |
| Cheese – Model 0 | −0.25 (−0.83, 0.34) | 0.412 |
| Cheese – Model 1 | −0.10 (−0.47, 0.28) | 0.609 |
| Cheese – Model 2 | −0.30 (−1.40, 0.79) | 0.588 |
| Cheese – Model 3 | −0.46 (−1.73, 0.81) | 0.476 |
| **FI** |  |  |
| Yoghurt – Model 0 | −0.01 (−0.04, 0.02) | 0.539 |
| Yoghurt – Model 1 | −0.03 (−0.06, 0.00) | 0.088 |
| Yoghurt – Model 2 | 0.00 (−0.08, 0.08) | 0.992 |
| Yoghurt – Model 3 | 0.04 (−0.05, 0.13) | 0.370 |
| Cheese – Model 0 | 0.00 (−0.04, 0.04) | 0.993 |
| Cheese – Model 1 | 0.02 (−0.02, 0.05) | 0.420 |
| Cheese – Model 2 | 0.00 (−0.09, 0.08) | 0.949 |
| Cheese – Model 3 | −0.01 (−0.10, 0.09) | 0.908 |
| **TUG** |  |  |
| Yoghurt – Model 0 | −0.01 (−0.03, 0.00) | 0.080 |
| Yoghurt – Model 1 | −0.01 (−0.03, 0.00) | 0.027 |
| Yoghurt – Model 2 | 0.00 (−0.03, 0.03) | 0.979 |
| Yoghurt – Model 3 | 0.01 (−0.02, 0.04) | 0.513 |
| Cheese – Model 0 | −0.01 (−0.03, 0.00) | 0.101 |
| Cheese – Model 1 | 0.00 (−0.02, 0.01) | 0.542 |
| Cheese – Model 2 | −0.02 (−0.05, 0.00) | 0.097 |
| Cheese – Model 3 | −0.01 (−0.04, 0.02) | 0.488 |
| **ADLs** |  |  |
| Yoghurt – Model 0 | −0.01 (−0.03, 0.01) | 0.418 |
| Yoghurt – Model 1 | −0.01 (−0.03, 0.01) | 0.325 |
| Yoghurt – Model 2 | −0.01 (−0.05, 0.04) | 0.745 |
| Yoghurt – Model 3 | 0.00 (−0.03, 0.03) | 0.921 |
| Cheese – Model 0 | −0.01 (−0.03, 0.02) | 0.714 |
| Cheese – Model 1 | 0.00 (−0.03, 0.02) | 0.848 |
| Cheese – Model 2 | 0.00 (−0.05, 0.05) | 0.985 |
| Cheese – Model 3 | 0.00 (−0.02, 0.03) | 0.818 |
| **Dichotomous outcomes** | | |
| **Low HGS vs Normal HGS** |  |  |
| Yoghurt – Model 0 | 1.02 (0.87, 1.18) | 0.767 |
| Yoghurt – Model 1 | 1.02 (0.87, 1.17) | 0.824 |
| Yoghurt – Model 2 | 1.48 (0.74, 2.65) | 0.220 |
| Yoghurt – Model 3 | 1.69 (0.81, 3.25) | 0.128 |
| Cheese – Model 0 | 0.98 (0.80, 1.17) | 0.810 |
| Cheese – Model 1 | 1.04 (0.85, 1.25) | 0.673 |
| Cheese – Model 2 | 1.35 (0.65, 2.48) | 0.373 |
| Cheese – Model 3 | 1.76 (0.84, 3.33) | 0.099 |
| **Frail/pre-frail vs Robust** |  |  |
| Yoghurt – Model 0 | 0.98 (0.88, 1.08) | 0.639 |
| Yoghurt – Model 1 | 0.93 (0.83, 1.03) | 0.174 |
| Yoghurt – Model 2 | 1.13 (0.75, 1.68) | 0.542 |
| Yoghurt – Model 3 | 1.49 (0.93, 2.37) | 0.087 |
| Cheese – Model 0 | 1.02 (0.90, 1.15) | 0.754 |
| Cheese – Model 1 | 1.08 (0.94, 1.23) | 0.286 |
| Cheese – Model 2 | 0.95 (0.59, 1.45) | 0.806 |
| Cheese – Model 3 | 0.91 (0.51, 1.57) | 0.753 |
| Estimates derived from multivariable linear and logistic regression models, as appropriate. Linear regression results are presented as β coefficients and logistic regression results as odds ratios (OR), each with 95% confidence intervals (CI). Model 0 is unadjusted; Model 1 adjusts for age and sex; Model 2 additionally adjusts for body mass index (BMI) and physical activity; Model 3 is fully adjusted for socioeconomic and lifestyle factors (education, household income, smoking status, and alcohol consumption). Abbreviations: TILDA, The Irish Longitudinal Study on Ageing; HGS, hand grip strength; FI, frailty index; TUG, Timed Up and Go; ADL, activities of daily living; CI, confidence interval. | | |

**Figure S1. Forest plot depicting association between yoghurt and cheese consumption with physical function in TILDA**. **A)** Continuous outcomes modelled with multivariate linear regression. **B)** Dichotomous outcomes modelled with multivariate logistic regression. TILDA, The Irish Longitudinal Study on Ageing; BMI, HGS, hand grip strength; FI, frailty index; TUG, Timed Up and Go; ADL, activities of daily living

**b**

**a**
